# Isolated auditory neuropathy at birth in congenital cytomegalovirus infection

**DOI:** 10.1186/s13052-019-0767-y

**Published:** 2020-01-06

**Authors:** Fabio Natale, Mario De Curtis, Bianca Bizzarri, Maria Patrizia Orlando, Massimo Ralli, Giuseppina Liuzzi, Barbara Caravale, Francesco Franco, Aurelia Gaeta, Antonella Giancotti, Francesca Yoshie Russo, Rosaria Turchetta

**Affiliations:** 1 Department of Maternal and Child Sciences and Urology, “Sapienza” University of Rome, Viale Regina Elena 324, 00161 Rome, Italy; 2 Department of Sense Organs, “Sapienza” University of Rome, Rome, Italy; 3 National Institute for Infectious Diseases (INMI) “Lazzaro Spallanzani”, Rome, Italy; 4 Department of Developmental and Social Psychology, “Sapienza” University of Rome, Rome, Italy; 5 Lazio Regional Health Authority, Rome, Italy; 6 Department of Public Health and Infectious Diseases, “Sapienza” University of Rome, Rome, Italy

**Keywords:** Congenital cytomegalovirus infection, Sensorineural hearing-loss, Auditory neuropathy, Auditory brainstem response, Otoacoustic emission

## Abstract

**Background:**

Congenital cytomegalovirus (cCMV) infection is the most frequent non-genetic cause of sensorineural hearing-loss (SNHL) (i.e., hearing loss due to a cochlear and/or auditory nerve damage). It is widely accepted that SNHL at birth, when associated to cCMV symptomatic infection involving the central nervous system, benefits from antiviral therapy started in the neonatal period. Conversely, there is no consensus for antiviral treatment in congenitally infected infants diagnosed with isolated SNHL (i.e., SNHL in an otherwise asymptomatic infant) at birth.

Our aim was to assess the frequency and the auditory outcome of isolated SNHL at birth due to auditory neuropathy (AN) (i.e., SNHL in a patient with normal cochlear function and auditory nerve dysfunction) in infants with cCMV infection.

**Methods:**

We retrospectively reviewed the clinical history of 60 infants, born at term, with cCMV asymptomatic infection, without additional risk factors for SNHL, and exhibiting bilateral “pass” otoacustic emissions (OAE). None of them underwent antiviral therapy.

Hearing thresholds were assessed by means of Auditory Brainstem Responses (ABR). AN affected children were followed up until possible normalization of the hearing thresholds or definitive diagnosis of AN. Each infant diagnosed with monolateral or bilateral AN was classified according to the worst ear threshold.

**Results:**

In our population, the first ABR was performed at a mean age of 5.00 ± 2.79 (SD) months and AN was diagnosed in 16/60 (26.67%) infants; in 4 infants the AN was defined as mild (4/4 monolateral), moderate in 11 (5/11 bilateral), and severe in 1 (bilateral). The mean age at first ABR was 3.69 ± 2.80 (SD) months in the 16 babies with AN and 5.48 ± 2.66 (SD) months in the 44 infants with normal hearing (*p* = 0.007). All AN cases spontaneously recovered a normal auditory threshold over time. The mean length of the audiological follow-up was 32.44 ± 17.58 (SD) months (range 5–60 months).

**Conclusion:**

A delayed maturation of the auditory pathways should be considered when a mild/moderate isolated AN at birth is detected in cCMV infected infants. Prospective studies conducted on larger populations, and with a longer audiological follow-up, are needed to confirm our findings.

## Background

Congenital cytomegalovirus (cCMV) infection is a leading non-genetic cause of sensorineural hearing-loss (SNHL) (i.e., hearing loss due to a cochlear and/or auditory nerve damage) and both sympotmatic and asymptomatic cCMV infections contribute to the burden of SNHL [1]. The benefits of antiviral therapy for SNHL at birth, when associated to cCMV symptomatic infection involving the central nervous system, is clearly demonstrated [2, 3]. The indications for therapy are less clear for the population of cCMV infected infants with isolated SNHL (i.e., SNHL in an otherwise asymptomatic infant) at birth: currently, it is not a clear consensus if these babies should start an antiviral therapy [4–6].

Hearing evaluation in infants is usually accomplished by means of Otoacustic Emissions (OAE), to investigate cochlear function, and Auditory Brainstem Responses (ABR). ABR testing is not only able to detect hearing loss associated with cochlear dysfunction but also forms of hearing loss due to problems in conveying the sound information to the brain [auditory neuropathy (AN)]. With AN, a newborn will pass OAE testing but fail ABR testing [7].

In this study, we retrospectively investigate the frequency and the outcome of isolated SNHL at birth due to AN in a selected population of infants with cCMV infection who did not receive antiviral therapy.

## Methods

### Study design

We retrospectively reviewed the prospectively collected data of a population of infants with cCMV infection, evaluated between January 2011 and June 2018 at the Outpatient Clinic of Congenital-Perinatal Infectious Diseases - Department of Maternal and Child Sciences and Urology - “Sapienza” University of Rome.

We included infants born at term (≥37 weeks gestational age) with a cCMV asymptomatic infection and an uneventful clinical course at birth. Namely, neonates suffering from diseases, or undergoing therapies, possibly affecting hearing outcome (e.g., asphyxia, severe hyperbilirubinemia, sepsis/meningitis, craniofacial malformation, chromosomal disorders, aminoglycosides and/or diuretic therapy) were excluded from the study. Other inclusion criteria were: 1) a bilateral “pass” OAE test in the first month of life; 2) a first ABR evaluation within the first 12 months of life (assuming that a SNHL detected in this time frame could be a congenital one), and 3) no antiviral therapy administered.

Maternal CMV primary infection was identified and dated according to serological CMV screening performed during pregnancy (CMV IgG seroconversion from negative to positive or low avidity CMV IgG together with CMV IgM antibodies). Congenital infection was established by means of viral culture (shell vial) or polymerase chain reaction performed on urine within the first 3 weeks of life. Asymptomatic infection was established according to widely agreed procedures [8]. IUGR infants, in absence of other signs of cCMV infection, were considered asymptomatic.

### Audiological assessment

All the infants underwent a first hearing screening within the first month of life (after birth, before hospital discharge, since 2015), by means of OAE. According to our protocol, infants with a “fail” result are referred within the first month of life to the Department of Sense Organs of our hospital to repeat OAE (Madsen Accuscreen ABR/TE/DP device-Otometrics A/S, Taastrup, Denmark) and perform ABR if a “fail” OAE result is confirmed. Infants with both a “fail” OAE and a lowered hearing threshold at ABR are candidates for antiviral treatment at our istitution. Infants with cCMV infection exhibiting a “pass” OAE are scheduled for ABR within 3 months of life and then every 6 months until the age of 2 years. After the age of two, the children are tested with play and pure tone audiometry. An otoscopic evaluation of tympanic membrane and of middle ear function trough tympanometry is performed during each follow-up visit.

ABR recordings were obtained while sleeping spontaneously and were performed with a GSI Audera device (Grason-Stadler, Eden Prairie, MN, USA) with a 21 stimuli per second click rate, in an analysis window of 12 msec. The highest intensity reached by this equipment is 100 dB-hearing level (dB-HL). The test implicates the repetition of the stimulus by 10 dB-HL steps, and the lowest intensity at which the V wave is observed, represents the hearing threshold of the subject. In accordance with other studies, our evaluation criterion only included the presence of the V wave [9, 10]. We did not evaluate wave V latency or amplitude values, since in newborns such values are highly variable even among normal hearing subjects, and reach adult values only at 2–3 years of age [11–13]. In all ABR measurements, hearing was classified as normal when the click-threshold was ≤30 dB-HL. Hearing loss was considered mild with threshold between 31 and 45 dB-HL, moderate between 46 and 70 dB-HL, severe between 71 and 90 dB-HL, and ≥ 91 dB-HL as profound hearing loss [14]. Each infant diagnosed with monolateral or bilateral AN was classified according to the worst ear threshold.

When the first ABR test was ≤30 dB-HL (normal), subsequent ABRs were not taken into account for the purpose of this study and the infant was categorized as NO-AN; whereas AN was diagnosed (hearing threshold > 30 dB-HL) at the first examination, subsequent ABR were considered until normalization of the hearing threshold (“maturative” AN) or definitive diagnosis of AN (“true” AN). Further ABRs after restoration of a normal auditory threshold were not considered to avoid a definition bias between fluctuations of the auditory threshold and a possible late onset SNHL. However, the auditory threshold at last audiological evaluation was recorded for each AN affected infant.

### Developmental assessment

Cognitive, language and motor areas of development were investigated with the Bayley Scales for Infant Development III Ed. (BSID-III) up to age 24 months by a single child neuropsychiatrist (BC). When more than one BSID-III test was available for the same child, the more recent evaluation was considered for the statistical analysis.

### Statistical analysis

Categorical data are expressed as numerical counts and percentages. The Fisher’s exact test was used for comparison between groups for categorical data. For continuous data, the Wilcoxon rank-sum (Mann-Whitney) test was used to compare mean ± standard deviation between groups. Results with a *P* value ≤0.05 were considered to be statistically significant. All analyses were performed with IBM SPSS Statistics for Windows, Version 22.0. Armonk, NY: IBM Corp.

## Results

One hundred-three children with cCMV infection were referred to our Outpatient Clinic of Congenital-Perinatal Infectious Diseases in the time interval considered. Thirty of them were excluded due to the presence of at least one exclusion criteria (cCMV symptomatic infection: 19 cases; prematurity: 5 cases; ototoxic medications: 3 cases; craniofacial malformation: 1 case; perinatal asphixia: 1 case; monolateral “fail” OAE: 1 case). Thirteen additional children were excluded due to missing audiological follow-up. The group eligible for the study consisted of 60 term infants with asymptomatic cCMV infection, all of them with a bilateral “pass” OAE. All babies were born to mothers who had suffered a primary CMV infection during pregnancy.

In our population, the first ABR was performed at a mean age of 5.00 ± 2.79 (SD) months. AN (any degree) was diagnosed in 16/60 (26.67%) infants [22/120 ears (18.33%)] at a mean age of 3.69 ± 2.80 (SD) months. This was significantly (*p* = 0.007) lower from the age at first ABR in the 44/60 infants (73,33%) with normal hearing [5.48 ± 2.66 (SD) months]. Demographical data of the study population were similar between AN and NO-AN groups (Table 1).

**Table 1:** Demographical data of the study population

Study population (60 infants) (%)	16 AN (26.67)	44 NO AN (73.33)	*p* value^a^
Timing of Maternal Infection			
• Periconceptional (%)	2 (12.50)	4 (9.09)	0.884^b^
• I Trimester (%)	3 (18.75)	12 (27.27)	
• II Trimester (%)	6 (37.50)	17 (38.64)	
• III Trimester (%)	5 (31.25)	11 (25.00)	
Male (%)	11/16 (68.75)	21/44 (47.73)	0.242^b^
Birth Weight (grams)	3317 ± 411^c^	3277 ± 457^c^	0.980^d^
Gestational age at birth (weeks)	38.69 ± 1.45^c^	38.75 ± 1.24^c^	0.830^d^

*AN* Auditory neuropathy. ^a^: *p* value ≤0.05 was considered to indicate statistical significance. ^b^: Fisher’s exact test. ^c^: Mean ± Standard Deviation. ^d^: Wilcoxon rank-sum (Mann-Whitney) test

According to the worst ear threshold, AN was classified as mild in 4 infants (4/4 monolateral), moderate in 11 (5/11 bilateral), and severe in 1 (bilateral) at baseline. In the sixteen AN patients, 22/32 (68.7%) ears were found to be affected by some degree of AN: mild in 8/32 (25.0%), moderate in 13/32 (40.6%), and severe in 1/32 cases (3.1%). No case of profound hearing loss was reported.

Further audiological evaluations were considered only in the 16 children diagnosed with AN at first ABR. The timing (mean ± SD) and the auditory performance of these infants, as detected by ABRs and audiometry over time, are illustrated in Table 2.

**Table 2:** Audiological results of infants diagnosed with AN from first ABR to last audiological evaluation. Mean age (months) ± standard deviation at evaluation are reported for each test

AN Patients n.	First ABR Right ear Left ear 3.69 ± 2.80 months		Second ABR Right ear Left ear 7.56 ± 2.87 months		Third ABR Right ear Left ear 11.83 ± 4.58 months		Fourth ABR Right ear Left ear 17.00 ± 2.83 months		Last audiological test 32.44 ± 17.58 months
1	70 dB	50 dB	70 dB	40 dB	40 dB	40 dB	**30 dB**	**30 dB**	Normal
2	50 dB	30 dB	**30 dB**	**30 dB**	–	–	–	–	Normal
3	30 dB	50 dB	**30 dB**	**30 dB**	–	–	–	–	Normal
4	40 dB	70 dB	**30 dB**	**30 dB**	–	–	–	–	Normal
5	40 dB	80 dB	30 dB	40 dB	**30 dB**	**30 dB**	–	–	Normal
6	40 dB	30 dB	**30 dB**	**30 dB**	**–**	**–**	–	–	Normal
7	50 dB	30 dB	50 dB	30 dB	**30 dB**	**30 dB**	–	–	Normal
8	40 dB	50 dB	30 dB	30 dB	**–**	**–**	–	–	Normal
9	40 dB	30 dB	**30 dB**	**30 dB**	**–**	**–**	–	–	Normal
10	50 dB	30 dB	40 dB	40 dB	**30 dB**	**30 dB**	–	–	Normal
11	50 dB	70 dB	70 dB	70 dB	**30 dB**	**30 dB**	–	–	Normal
12	50 dB	40 dB	**30 dB**	**30 dB**	–	–	–	–	Normal
13	40 dB	30 dB	50 dB	40 dB	50 dB	70 dB	40 dB	30 dB	Normal
14	50 dB	30 dB	**30 dB**	**30 dB**	–	–	–	–	Normal
15	30 dB	50 dB	**30 dB**	**30 dB**	–	–	–	–	Normal
16	40 dB	30 dB	**30 dB**	**30 dB**	–	–	–	–	Normal

*AN* Auditory neuropathy, *ABR* Auditory Brainstem Response, *dB* Decibel normal hearing level. Normal hearing thresholds are highlighted in bold characters

**Fig. 1:**
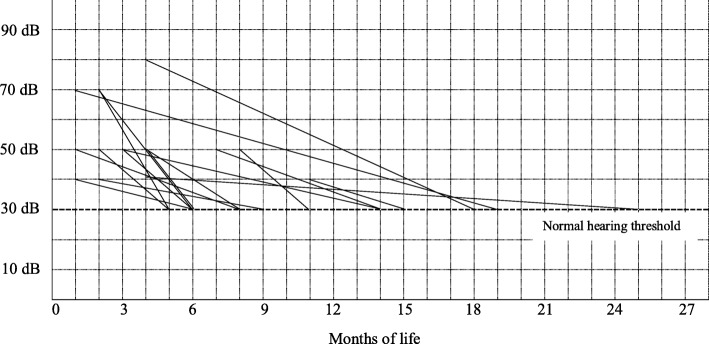
Temporal variations of the auditory thresholds in children diagnosed with AN (according to the worst ear threshold). AN: auditory neuropathy; dB: decibel normal hearing level

The temporal variations of the auditory thresholds, from AN detection to normalization, for each of the 16 children, are detailed in Fig. 1.

All children diagnosed with AN at baseline achieved a normal auditory threshold (≤30 dB-HL) over time; in 14/16 cases (87,50%) this was accomplished within 18 months of life. In only 1 case (n.13, Table 2) the normalization of the auditory threshold was established after 2 years of life with pure tone audiometry (Fig. 1). In 3 cases (n.10, 11, and 13), a fluctuation of the hearing threshold was detected by means of ABR before normalization (Table 2).

At follow up, the last audiological evaluation was accomplished by means of play and pure tone audiometry in 13/16 (81.25%) infants aged ≥ 2 years and was normal in all cases (Pure Tone Average ≤ 25 dB-HL); in the remaining 3/16 (18.75%) infants aged < 2 years the last audiological evaluation coincided with the last normal ABR. The mean age at last audiological evaluation was 32.44 ± 17.58 (SD) months (range 5–60 months) in AN infants.

When the cognitive, language, and motor areas were investigated by means of BSID-III, no significant differences were detected between groups (Table 3).

**Table 3:** BSID III evaluation of the study population

Study population (60 infants) (%)	16 AN (26.67)	44 NO AN (73.33)	*p* value^a^
Tested infants (%)	13/16 (81.25)	37/44 (84.09)	1.000^b^
Age at last BSID-III (months)	19.46 ± 7.29^c^	12.11 ± 6.92^c^	0.009^d^
Composite Cognitive score	106.92 ± 9.69^c^	105.27 ± 9.20^c^	0.662^d^
Composite Language score	98.92 ± 8.55^c^	104.03 ± 9.81^c^	0.093^d^
Composite Motor score	100.31 ± 9.10^c^	95.27 ± 9.42^c^	0.117^d^

*BSID III* Bayley Scales of Infant Development III Edition. *AN* Auditory neuropathy. ^a^: *p* value ≤0.05 was considered to indicate statistical significance. ^b^: Fisher’s exact test. ^c^: Mean ± Standard Deviation. ^d^: Wilcoxon rank-sum (Mann-Whitney) test

## Discussion

cCMV infection is the most common non-genetic cause of SNHL, whereas the term SNHL classically defines a hearing loss possibly due to both cochlear and/or auditory nerve damage.

Two recent consensus statements on cCMV infection have recently highlighted the uncertainty surrounding the therapeutic management of isolated SNHL at birth [4, 5]. In fact, despite some experts believe that affected children should be treated with antiviral drugs [5, 6], no definitive evidence about the potential benefit of antivirals is currently available [4].

The aim of our study was to investigate the auditory evolution of children with cCMV asymptomatic infection, not receiving antiviral treatment, who had a diagnosis of isolated SNHL at birth due to AN (i.e., abnormal/absent ABR response in combination with a bilateral “pass” OAE). In order to emphasize any change in the auditory threshold over time, we choose to rely on the worst ear for severity grading of SNHL, rather than on a functional evaluation based on best-ear assessment [2].

Our study demonstrates that a mild/moderate isolated AN at birth (particularly in the range ≤ 50 dB-HL), is a not negligible event in infants with asymptomatic cCMV infection. Of interest, a normal hearing threshold was always recovered over time, in absence of antiviral treatment, in these cases.

Our data suggest that a delayed maturation of the auditory pathways, rather than a “true” SNHL, may affect the auditory outcome of the infants diagnosed with AN. This hypothesis is supported by the existence of a difference in the mean age at first ABR between the two study groups, whereas AN infants underwent first ABR significantly earlier (*p* = 0.007) than not affected ones. In this light, also the distribution of AN cases along all trimesters of maternal infection (Table 1) suggests that a different pathogenesis is involved in these cases if compared to SNHL cases reported in medical literature, where a primary maternal infection of the first trimester of pregnancy is demonstrated to be a major risk factor [15, 16].

The presence of a physiological delay of maturation of the auditory pathways in newborns and children has already been highlighted in previous studies, with consequences on the results of ABR and cortical auditory evoked potential [17, 18]. Studies performed in neonates and infants at risk of hearing loss have shown the possibility of complete or partial recovery of auditory thresholds over time, even in severe cases [9, 10]. Interestingly, in a large group of infants at risk of hearing loss with abnormal ABR at initial hearing evaluation, a normal OAE at first evaluation and a very young age at initial hearing screening were associated with a normal ABR threshold restoration on reexamination, and a delayed myelination of the acoustic pathway was deemed the main reason for this to occur [9]. Oligodendrocyte maturation - and then myelin production - depends on a variety of growth factors, hormones, cytokines, surface receptors, and secreted ligands [19]; in this light, the proinflammatory state which follows in utero CMV infection might affect, to some extent, the maturation of the auditory pathway [20]. However, due to the lack of a control group of term, not-infected infants, we were not able to establish the real contribution of cCMV infection to the incidence of isolated AN in our population.

Several studies have investigated the auditory evolution of isolated SNHL at birth in cCMV infection [21–23]. Dahle et al [21], have demonstrated that an improvement in hearing thresholds may occur in up to 47.9% of infants with cCMV asymptomatic infection. Foulon et al [22], have reported a complete normalization of the auditory thresholds in 35.0% of the infants with cCMV asymptomatic infection. Recently, Pasternak et al [23], in a retrospective study on 59 infants with cCMV infection and isolated SNHL who received prolonged antiviral treatment, reported an improvement of 68.6% of affected ears with hearing deficit at baseline during follow-up (96.3% returning to normal hearing). In the studies mentioned above [21–23], the hearing thresholds were investigated by means of ABR and no relationship with OAE results was established; then, what part of SNHL burden was attributable to AN is unknown in these studies.

Our results are in accordance with Foulon et al [24], that among 18 hearing-impaired children out of a cohort of 206 cCMV infected infants, could not detect OAE in any of the SNHL cases; the authors concluded that AN does not appear to be a feature of cCMV infection. Royackers et al [25], describe only one case (in seventy cCMV infected children) of AN in a symptomatic infection that spontaneously resolved over time.

When the cognitive, language, and motor areas were investigated by means of BSID-III composite scores, no significant differences were detected between the two groups (Table 3), suggesting that a delay of the auditory pathway maturation does not seem to affect the child development. The validity of these results could be partially flawed by significantly different lenghts of Bayley III follow up between the two groups (Table 3); a greater concern for parents of children diagnosed with SNHL may explain the greater compliance with the scheduled Bayley III assessment in this group.

Although based on a small number of cases, the strength of our work is represented by the highly selected population included, avoiding biases due to prematurity and additional diseases/therapies known to affect the audiologic outcome.

However, several limitations should be noted. This is a retrospective study, thus dealing with possible biases (mostly related to information and/or selection bias) relevant to the nature of the study itself. Further, though our population was scheduled for ABR within 3 months of life, the resulting mean age at first ABR was substantially higher (5.00 ± 2.79 months). Frequent postponements of audiological appointments by parents and busy waiting lists were the main reasons for this to occur. Finally, the lack of a systematic ABR assessment in the first month of life prevented us an accurate estimate of the burden of AN at birth and, most important, some applicability of these data in the restricted time window available to decide if therapy has to be performed (within 28 days of life) [2, 3]. Nonetheless, the sixteen AN cases were diagnosed at a mean age of 3 months in our study, thus suggesting a congenital AN form be involved in most, if not all of these cases.

Despite the above limitations, we believe that this study could raise some important cues worthy of being further verified in prospective, possibly controlled, trials.

Infants with mild/moderate SNHL on first ABR and a bilateral pass OAE should be suspected for a maturation delay of the auditory thresholds and, in our opinion, these infants should be possibly retested before any possible therapeutic decision. Severe/profound AN cases were almost absent in our cohort (only 1 ear with severe AN) and this prevented us any meaningful consideration about the possible management of AN of higher degree.

OAE, together with ABR, should be part of the initial hearing evaluation of SNHL in cCMV infected infants in order to assess if a cochlear damage, or an auditory neuropathy, is responsible for a hearing loss. In fact, if the clinician relies exclusively on ABR testing to assess the burden of SNHL at birth, this might include, according to our experience, a variable amount of mild/moderate “maturative” AN cases possibly leading to antiviral overtreatment in this population.

Besides, testing cCMV infected infants with both OAE and ABR could allow a separate evaluation of both audithory outcome (SNHL due to cochlear damage vs. SNHL due to AN), thus enabling to draw data about a possible, different management between the two types of hearing loss.

## Conclusions

In cCMV infected infants, a delayed maturation of the auditory pathways should be suspected when a mild/moderate isolated AN at birth is detected. It might be advisible to retest these infants before any possible therapeutic decision.

Prospective, possibly controlled trials, conducted on larger populations and with a longer audiological follow-up, are needed to confirm our findings.

## Data Availability

The datasets used and/or analysed during the current study are available from the corresponding author on reasonable request.

## Abbreviations

ABR: Auditory brainstem response
AN: Auditory neuropathy
BSID-III: Bayley Scales for Infant Development III Edition
cCMV: Congenital cytomegalovirus
dB-HL: Decibel-hearing level
OAE: Otoacustic emissions
SD: Standard deviation
SNHL: Sensorineural hearing loss
